# Human induced pluripotent stem cells and organoid models in space for investigating severe pediatric metabolic and congenital disorders

**DOI:** 10.1093/pcmedi/pbag028

**Published:** 2026-09-29

**Authors:** Sara Benedetti, Hana Štupica, Gian Vincenzo Zuccotti, Volkmar Weissig, Davide Marotta, Carlo Pappone, Alba Tull, Kathleen H Rubins, Afshin Beheshti, Christopher E Mason, Simona Ferraro

**Affiliations:** Arrhythmia and Electrophysiology Centre, IRCCS Policlinico San Donato, San Donato Milanese, Milan 20097, Italy; Center for Neurogenomics and Cognitive Research, Vrije Universiteit Amsterdam, 1081 HV Amsterdam, The Netherlands; Department of Pediatrics, Buzzi Children’s Hospital, Milan 20154, Italy; Department of Biomedical and Clinical Science, University of Milan, Milan 20157, Italy; Department of Pharmaceutical Science, Midwestern University College of Pharmacy Glendale, Arizona, AZ 85308, United States; Helogen Corporation, New York, NY 10013, United States; School of Biomedical Engineering, University of Technology Sydney, Sydney, NSW 2007, Australia; Arrhythmia and Electrophysiology Centre, IRCCS Policlinico San Donato, San Donato Milanese, Milan 20097, Italy; Department of Cardiology, Electrophysiology, and Cardiac Pacing Unit, Vita-Salute San Raffaele University, Milan 20132, Italy; Institute for Molecular and Translational Cardiology (IMTC), IRCCS Policlinico San Donato, San Donato Milanese, Milan 20097, Italy; Department of Biological Sciences, Carnegie Mellon University, Pittsburgh, PA 15213, United States; National Aeronautics and Space Administration, Johnson Space Flight Center, Houston, TX 77058, United States; Center for Space Biomedicine, Trivedi Institute for Space and Global Biomedicine, University of Pittsburgh, Pittsburgh, PA 15219, United States; Department of Computational and Systems Biology, University of Pittsburgh, Pittsburgh, PA 15219, United States; Stanley Center for Psychiatric Research, Broad Institute of MIT and Harvard, Cambridge, MA 02142, United Sttes; McGowan Institute for Regenerative Medicine, Department of Surgery, University of Pittsburgh, Pittsburgh, PA 15219, United States; Center for Space Biomedicine, Trivedi Institute for Space and Global Biomedicine, University of Pittsburgh, Pittsburgh, PA 15219, United States; The HRH Prince Alwaleed Bin Talal Bin Abdulaziz Alsaud Institute for Computational Biomedicine, Weill Cornell Medicine, New York, NY 10021, United States; The WorldQuant Initiative for Quantitative Prediction, Weill Cornell Medicine, New York, NY 10065, United States; Department of Physiology and Biophysics, Weill Cornell Medicine, New York, NY 10065, United States; Department of Pediatrics, Buzzi Children’s Hospital, Milan 20154, Italy

**Keywords:** organoids, hiPSC, 3D cell culture, rare diseases, congenital heart defect, mitochondrial disorder

## Abstract

As biomedical research in space expands, microgravity and spaceflight-associated stressors are revealing cellular responses that may provide new insights into rare and severe metabolic and congenital disorders. Human induced pluripotent stem cells (hiPSCs) and organoids are particularly promising as they enable patient-specific modelling in the space environment. In this review, we examine the current literature on space-based hiPSC experimental models, highlighting their relevance to pediatric disorders with well-defined genetic backgrounds, namely mitochondrial diseases and congenital heart disorders. We summarize the evidence that spaceflight conditions alter mitochondrial function, cytoskeletal organization, oxidative stress, calcium handling, cellular maturation, and gene expression, and discuss how these responses may converge with metabolic disease-associated phenotypes. We highlight both adaptive and pathological responses reported in space-based hiPSC studies and identify key experimental and mechanistic gaps. Finally, we consider how advances in stem cell technologies and space-based biomedical research may enable the development of personalized, patient-specific disease models and therapeutics for rare pediatric diseases, ultimately accelerating translational discovery and clinical innovation.

## Introduction

With advancements in technology and personalized medicine, biomedical research has relied on experimental cell models to understand underlying disease mechanisms, mimic human physiology and pathology, and develop novel therapeutic interventions. Prior work has highlighted human induced pluripotent stem cells (hiPSCs) and complex 3D organoids to model a wide array of diseases. These techniques overcome several challenges, including small patient populations, geographic dispersion of affected individuals, ethical constraints associated with research involving vulnerable populations such as children, and the shortcomings of animal models. While cell models enable detailed studies of underlying cellular and molecular mechanisms, 3D organoids better mimic the structure and function of cells and tissues, providing a more accurate representation of the disease environment [1]. Therefore, such approaches offer significant benefits for studying rare and severe pediatric conditions like mitochondrial diseases and congenital heart disorders (CHDs) where obtaining primary cells directly from patients is difficult [2]. Both diseases are extremely heterogeneous, making patient-specific models essential for understanding their disease mechanisms and development. For instance, the benefits of shifting from rodent models to hiPSCs have already been visible in the mitochondrial disorders, where *SURF1* knock-out (KO) mice fail to develop pathological features of Leigh syndrome, while isogenic hiPSC lines carrying *SURF1* mutations in healthy or patient-specific backgrounds and cerebral organoids derived from these lines showed compromised neuronal morphogenesis [3].

In recent years, stem-cell-based models have been extensively used in space research to explore various biological processes and potential therapeutic applications. Notably, mitochondrial dysfunction and metabolic alterations have emerged as a consistent phenotype of spaceflight. Multi-omics analysis of astronauts across multiple missions has revealed progressive impairment of mitochondrial oxidative phosphorylation (OXPHOS) during spaceflight, with delayed recovery post-return [4, 5]. Given the central role of mitochondrial metabolism changes in mitochondrial diseases and CHDs, these multi-omics findings provide a strong rationale for investigating mitochondrial diseases and CHDs in the space environment [6, 7]. Thus, space-based models could offer a powerful platform to study how metabolic perturbations influence the mechanistic pathways in these two disorders.

hiPSCs are particularly suited for this approach as they can be generated from specific patients and astronauts with known genotypes and clinically detected phenotypes and differentiated into disease-relevant cell models [5]. For example, cortical and dopaminergic (DA) organoids cultured in low Earth orbit (LEO) have been shown to be characterized by an imbalance between proliferation- and maturation-associated gene expression, suggesting that cells matured more quickly in LEO [8]. This effect may be especially valuable for hiPSC-based models, since the immature phenotype of differentiated cells and organoids remains one of the major limitations in modeling the disease. Moreover, the space environment may serve not only as a platform for accelerated growth but also as a biologically distinct stress context that can unmask disease-associated phenotypes, accelerate stress responses, and potentially enhance features difficult to observe under terrestrial conditions.

This paper examines how experimental cell models cultured under spaceflight conditions can advance our understanding of rare and severe pediatric disorders, particularly mitochondrial diseases and CHDs. We review current evidence, discuss key opportunities and challenges, and the potential of space-based biomanufacturing to uncover disease mechanisms and improve diagnostics, supporting the development of novel therapies (Fig. 1).

**Figure 1:**
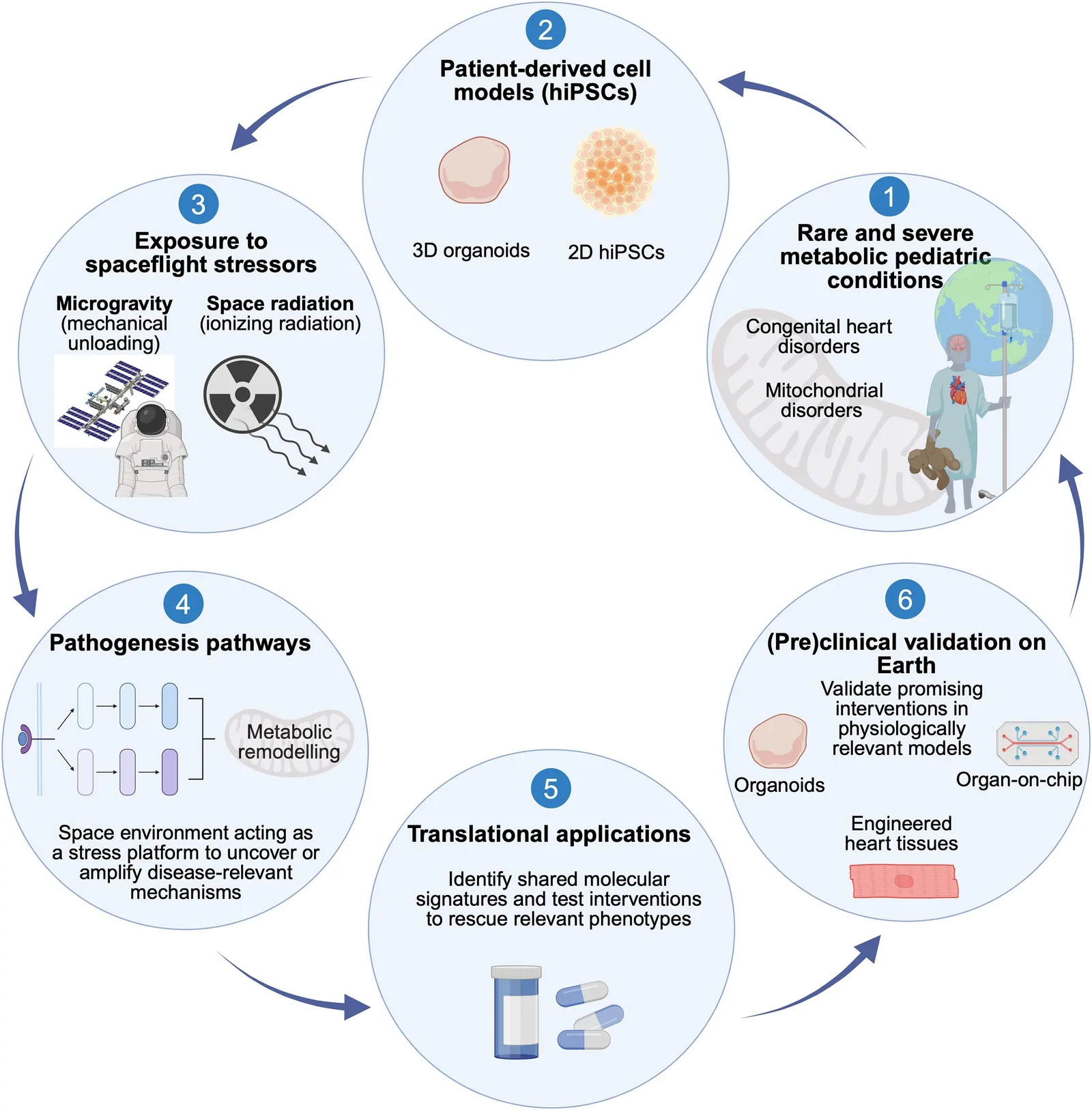
Integrative framework for the use of patient-derived hiPSC models to investigate rare metabolic disorders and support therapeutic translation. Patient-derived hiPSCs and 3D organoids from children with rare metabolic disorders, such as mitochondrial diseases and CHDs, could be exposed to spaceflight stressors, including microgravity and space radiation. The space environment could act as a cellular stress platform, potentially revealing novel disease mechanisms or amplifying disease-associated vulnerabilities and molecular pathways that are less apparent under terrestrial conditions. Metabolic signatures shared between space-exposed models and patients on Earth could subsequently be used to identify potential therapeutic targets and candidate interventions. Promising findings could then be validated in physiologically relevant terrestrial models, followed by conventional preclinical and clinical studies. Ultimately, this framework could contribute to the development of therapeutic strategies for patients with rare and severe metabolic pediatric disorders. Created with Biorender.com.

## Organoids and hiPSC-based models for rare mitochondrial disorders on Earth

### Mitochondrial disorders

Mitochondrial diseases comprise a genetically and clinically heterogeneous group of congenital or acquired disorders of mitochondrial energy metabolism and functional disruption, frequently affecting several organs, resulting in a large variety of phenotypes [9]. These disorders often affect high-energy-demanding tissues such as brain, muscles and heart, leading to severe phenotypes including myopathies, neurodegeneration, and multi-system failure [9]. Inherited or *de novo* mutations in >350 genes encoded by either mitochondrial DNA (mtDNA) or nuclear DNA (nDNA, hosting mitochondrial genes), have been identified to cause mitochondrial diseases [10]. Diseases caused by malfunctioning mitochondria as a result of either mtDNA or nDNA mutations are classified as “primary mitochondrial diseases” in contrast to mitochondrial disorders of non-genetic origin, which are referred to as “secondary mitochondrial diseases” [11].

Proteins essential for mitochondrial function, whose correct expression is affected by mutations, may broadly be categorized into three groups. First, all proteins and polypeptides that are components of the respiratory chain, comprising proteins needed for the assembly of the OXPHOS complexes, including co-factors. Second, all enzymes catalyzing metabolic pathways that are at least partially located inside the mitochondrial matrix. Third, all enzymes and proteins responsible for mitochondrial quality control (MQC). The latter refers to a system comprised of three major biological processes essential for maintaining a fully functioning population of mitochondria. MQC involves continuous mitochondrial biogenesis, removal of dysfunctional organelles via mitophagy, and maintenance of a proper balance between fission and fusion of the mitochondrial network [12]. Any disturbance of the MQC will eventually result in an accumulation of malfunctioning mitochondria over time, thereby contributing to the pathogenesis of age-related diseases [13].

The most commonly shared feature of all mitochondrial diseases is the suboptimal supply of adenosine triphosphate (ATP), which is the main source of energy for the body. Consequently, tissues with high energy demand (metabolically active tissues), such as central nervous system, heart, kidney, liver, and skeletal muscles are particularly vulnerable to mitochondrial dysfunction [9]. Clinical symptoms caused by mitochondrial malfunction can emerge at any age, yet pediatric mitochondrial diseases are generally more serious than adult-onset. Symptoms may span from acute life-threatening incidents to recurrent episodes with partial recovery to slow but progressive neurodevelopmental deterioration [9].

Leigh syndrome represents a prominent example of a severe pediatric mitochondrial disease in which mitochondrial metabolism impairments predominantly affect the central nervous system. Its progressive mitochondrial encephalopathy with initial symptoms appearing as early as 7 months of age, leads to death at a median age of 2.4 years and affects an estimated 1 in 40 000 individuals [14]. Dysfunction of cerebral mitochondrial respiration resulting in ATP depletion is generally accepted as the major cause of its neuropathology, originally described >75 years ago [14]. In postmortem pathological specimens, Leigh observed focal and bilateral lesions in the basal ganglia, thalamus, and brain stem regions, leading to general developmental regression, early ophthalmic signs such as retinal dysfunction and ophthalmoplegia, feeding difficulties, and vomiting. As the disease progresses, ataxia, seizures, and dystonia become typical, with death eventually caused by brain stem failure [14]. More than 100 genetic mutations affecting mtDNA and nDNA have been associated with Leigh’s disease so far, affecting multiple components of mitochondrial energy metabolism, with respiratory chain complex I representing one of the most affected pathways [15].

Overall, mitochondrial diseases showcase how genetic and clinical heterogeneity can converge on impaired energy metabolism, whilst still producing different tissue-specific manifestations. This genetic complexity, together with limited accessibility of patient tissues highlights the need for patient-specific cellular models capable of recapitulating disease-relevant cell types.

### hiPSC models of mitochondrial disorders

Many different model systems have been created to understand mitochondrial disease mechanisms, ranging from yeast, fruit flies (*Drosophila melanogaster*), and nematode roundworms (*Caenorhabditis elegans*) to mammalian models such as the *Ndufs4* KO mouse model of complex I deficiency [16]. However, these systems have shown poor translation into clinical applications. As a complementary alternative, more recent models are often based on patient-derived hiPSCs, which can be used to study disease-relevant cell types while preserving the patient’s genetic background, including cardiomyocytes and neurons, and to generate 3D organoids [15]. Such hiPSC-based patient-specific disease models also open up avenues for the development of high-throughput drug screens and subsequently for personalized treatments of mitochondrial disorders.

Leigh syndrome provides a relevant example of the advantages of hiPSCs. Although its *Ndufs4* KO model remains one of the most widely used animal models of complex I-deficient Leigh syndrome, it represents only one genetic cause of the disease, specifically loss of *NDUFS4* function. Mouse (sub)strain selection can also influence the resulting phenotype, potentially confounding the interpretation of experimental results [16]. In contrast, patient-derived hiPSCs enable disease-associated mutations to be studied directly in human cells with the patient’s genetic background. For example, hiPSC-derived neural progenitor cells (NPCs) carrying the m.9185T > C mutation in the *MT-ATP6* gene, a recurrent cause in Leigh syndrome [17], exhibited disease-relevant cellular phenotypes, including decreased ATP production, increased mitochondrial membrane potential, and altered calcium (Ca^2+^) homeostasis [18]. HiPSC-derived neurons and cardiomyocytes have also been extensively used to model other mitochondrial diseases, such as mitochondrial encephalopathy, lactic acidosis, and stroke-like episodes and Barth syndrome (BTHS). These models capture hallmark mitochondrial dysfunctions, such as impaired OXPHOS, elevated reactive oxygen species (ROS), and metabolic reprogramming [15].

Recent advances in mitochondrial hiPSC modelling have further increased their experimental value. The generation of isogenic controls using CRISPR/Cas9 allows for generating a disease-relevant mutation while the remaining genetic background is preserved. For instance, CRISPR-corrected hiPSC lines from patients with POLG-related disorders, where *POLG* encodes a subunit of mitochondrial DNA polymerase γ, which is essential for mtDNA replication and repair, have been used to identify defects in mtDNA replication and bioenergetics in neural progenitor cells [19]. Similarly, advances in metabolic flux analysis and live-cell imaging have improved the real-time assessment of mitochondrial function in hiPSC-derived neurons and organoids.

The development of 3D organoid models further expands the ability of hiPSCs to reveal tissue-specific phenotypes of mitochondrial diseases. Cerebral organoids generated from mitochondrial disease hiPSCs have shown aberrant neurodevelopmental patterns, increased apoptosis, and altered neuronal network activity, suggesting that mitochondrial dysfunction can affect neurological development before the manifestation of neurodegeneration [20]. Likewise, cardiac organoids derived from patients with *TAZ* mutations associated with BTHS exhibit contractile dysfunction and abnormal mitochondrial ultrastructure, making them valuable models for therapeutic screening [15].

These data show how patient-derived hiPSCs, isogenic gene-edited controls, and 3D organoid models provide complementary tools for linking the mitochondrial mutations to a specific human genetic background. Notably, differentiation of patient-derived hiPSCs into different cell types also helps elucidate how a single mitochondrial defect can produce distinct tissue-specific manifestations, addressing one of the major challenges in understanding clinically heterogeneous mitochondrial diseases.

## hiPSC-based models for congenital heart disorders on Earth

CHDs are structural abnormalities of the heart or great vessels occurring in ∼1% of births and are among the most common and serious birth defects [21]. Indeed, CHDs remain one of the leading causes of infant death associated with several co-morbidities and arrhythmias [22]. Approximately 20% of CHD cases are syndromic and are associated with extracardiac anomalies, such as neurological or skeletal defects, as observed in Noonan and Holt-Oram syndromes [23]. Although CHDs are generally considered multifactorial, ∼20% of total CHDs can be linked to a known genetic cause, including trisomy 21 (10% of syndromic CHD), 22q11 deletion in DiGeorge syndrome (DGS) (2%), and single gene defects (30%), involving at least 130 genes [24]. Besides its prominent genetic causes, accumulating evidence suggests that altered mitochondrial function and energy metabolism can strongly contribute to CHD pathophysiology [6].

Due to the genetic heterogeneity of CHDs and difficulty of directly observing early human cardiac development, it remains challenging to understand how individual genetic defects give rise to structural cardiac abnormalities and how these developmental defects interact with cellular metabolism. Since their first description in 2008, hiPSCs offer the chance to study disease mechanisms at the cellular level within a patient-specific genetic background and provide an opportunity to test novel therapeutic interventions. Below we review several relatively common CHDs and illustrate how the available hiPSC-based cellular models have contributed to understanding their underlying cellular and developmental mechanisms. Because of the heterogeneity in CHDs, we present them in terms of their mechanistic processes, namely, metabolic/mitochondrial dysfunction, structural genetic defects, sarcomeric mutations, and calcium-handling abnormalities.

### Metabolic and mitochondrial abnormalities

Mitochondria occupy ∼20%–40% of the volume of adult cardiomyocytes and represent the major source of cardiac energy, generating ∼95% of the ATP consumed by the heart [25, 26]. During cardiomyocyte maturation, mitochondria undergo extensive metabolic remodeling, characterized by transition from predominantly glycolytic metabolism towards more efficient mitochondrial OXPHOS, with fatty-acid becoming the major energy substrate [25]. Consequently, mitochondrial function is important not only for ATP production but also for cardiomyocyte differentiation and maturation.

Mitophagy, clearance of damaged and immature mitochondria, is one of the most important processes during cardiac maturation as it facilitates mitochondrial turnover and metabolic reprogramming toward the mature oxidative phenotype [25]. Experimental disruption of mitophagy was shown to impair mitochondrial maturation and metabolic reprogramming, ultimately resulting in lethal cardiomyopathy [27]. Mitochondria also regulate cardiomyocyte survival through the intrinsic apoptotic pathway. Oxidative stress and mitochondrial damage can lead to the release of pro-apoptotic factors, including cytochrome c, thereby activating downstream apoptotic pathways leading towards cardiomyocyte death [6]. The importance of mitochondrial function in cardiac pathology also comes from mitochondrial disorders, in which cardiac involvement is common and can manifest as cardiomyopathy and arrhythmias [28].

Patient-derived hiPSC cardiomyocytes (hiPSC-CMs) provide a valuable platform to investigate how these mitochondrial abnormalities translate into human cardiac phenotypes. Studies using cardiac patient hiPSC-CMs consistently display defective mitochondrial respiration, oxidative stress, increased apoptosis, and failed antioxidant response [29, 30]. Furthermore, hiPSC-CMs with impaired mitochondrial fatty acid oxidation showed abnormal Ca^2+^ handling and repolarization, directly changing cardiac electrophysiology [31]. Together, these findings demonstrate that mitochondrial abnormalities play an important role in diverse CHDs. However, the causative role of mitochondrial dysfunction in these cardiac phenotypes is often unclear. Novel patient-derived hiPSCs could more precisely determine whether mitochondrial processes, such as mitophagy, OXPHOS, or mtDNA maintenance precedes the development of CHDs and potentially identify novel therapeutic targets.

### Structural and developmental defects: tetralogy of Fallot and hypoplastic left heart syndrome

#### Tetralogy of Fallot

Tetralogy of Fallot (TOF) is a heart conotruncal defect, representing the most common CHD, affecting ∼0.3 children per 1 000 live births [32, 33]. TOF is caused by abnormal signaling during the early stages of embryogenesis, leading to alignment defects of the heart outflow tract. Although surgical correction has substantially improved survival, cardiac arrhythmia constitutes an important cause of mortality in TOF patients.

About 20% of TOF cases can be attributed to chromosomal abnormalities, including 21, 13, 18 trisomies and X chromosome monosomy; 16% TOF patients carry 22q11 microdeletion causing DGS, associated with haploinsufficiency of the *TBX1* transcription factor which plays an important role in cardiac development. Several rare variants have been identified in patients affected by isolated TOF affecting genes encoding cardiac transcription factors like *NKX2–5* (mutated in ∼4% of TOF patients), *GATA4, TBX5*, and *HAND2* [34, 35]. Due to this genetic heterogeneity, patient-specific models are deemed particularly valuable for determining how different genetic backgrounds lead to similar structural cardiac phenotypes.

A recent study demonstrated the potential of hiPSCs by directly comparing hiPSC-CMs from patients with syndromic DGS-associated TOF and nonsyndromic TOF [35]. This study suggested that developmental defects leading to DGS-TOF are more complicated than just *TBX1* haploinsufficiency. Indeed, impaired ventricular specification and downregulated cardiac gene expression were evident only in hiPSC-CMs from DGS-TOF patients. These cells also exhibited altered electrophysiological characteristics leading to predisposition to ventricular arrhythmias, thus providing an explanation for sudden cardiac death occurring in DGS patients even in the absence of cardiac malformations.

Despite the advantages of using hiPSC-CMs, they have important limitations for studying structural CHDs. First, the heterogeneity of ventricular expression of cultured hiPSC-CMs may affect electrophysiologic assessment. In addition, *in vivo* cardiac development and morphogenesis cannot be replicated using this *in vitro* model, since temporal and spatial interaction between different types of cells is crucial in TOF pathogenesis. This is especially relevant for TOF, which is based on cardiac morphogenesis abnormalities rather than an isolated cardiomyocyte defects.

Recently, self-organising structures called “cardioids” that develop chamber-like structures containing a central cavity have been derived from hiPSCs, which may provide a better model to study defective cardiogenesis in TOF-DGS by better reproducing early cardiac morphogenesis [36]. These models could help to study how the synchronized expression of different genes encoding transcription factors like TBX5, together with ion channels, and structural and sarcoplasmic reticulum proteins finely regulate differentiation and morphogenesis.

Beyond the electrophysiological and structural defects, metabolic defects seem to also play an important role in the cardiac phenotype of TOF. Transcriptomic and metabolic analyses have identified mitochondrial metabolism-related pathways among the major pathways altered in TOF, with the largest changes seen in genes related to mitochondrial ATP production and fatty acid metabolism [37]. Consistently, patients with TOF were also shown to have impaired OXPHOS and mitochondrial respiratory chain complex I, I + III and IV cytochrome content and ATPase activity [38]. These data suggest that mitochondrial metabolic remodeling could contribute to the myocardial phenotype alongside its underlying structural abnormalities. Patient-derived hiPSC-CMs could be used to determine whether these metabolic alterations arise from the genetic background of TOF or develop as a secondary response to altered hemodynamics.

#### Hypoplastic left heart syndrome

Hypoplastic left heart syndrome (HLHS) is the most severe form of CHD, with 30% mortality from heart failure in the first year of life, and it is characterized by hypoplasia of the left ventricle and other left-side heart structures [39]. Despite advances in early surgical procedures, long-term outcomes often remain poor, therefore requiring a better understanding of the pathogenetic mechanisms. Two leading theories have been postulated: either hypoplasia is a consequence of altered hemodynamics or HLHS may be a form of primary cardiomyopathy. Despite many efforts, the underlying genetic causes remain elusive, suggesting a multifactorial etiology of HLHS. Defects in genes including *NKX2-5, NOTCH1, ETS1*, and *HAND1* have been associated with cardiomyocyte hyperplasia during early development, leading to a disruption of the balance between different layers of the ventricular wall [40].

Patient-derived hiPSC models have provided important evidence in support of intrinsic cardiomyocyte abnormalities in HLHS. Jiang *et al*. demonstrated impaired differentiation of hiPSC-CMs from HLHS patients: hiPSC-CMs showed lower beating rates and accelerated Ca^2+^ transient decay, associated with ryanodine receptor (RyR2) and sarcoplasmic reticulum dysfunction, suggesting an immature differentiation process [41]. Kobayashi *et al*. further showed decreased levels of *NKX2–5, HAND1/2, TBX2, and NOTCH/HEY* signaling in HLHS-hiPSC-CMs, supporting impaired cardiomyogenic differentiation [42].

More recently, hiPSC-CMs have also revealed a potential contribution of mitochondrial metabolism to the dysfunctions seen in HLHS. Contractile force and acceleration were significantly reduced in hiPSC-CMs derived from HLHS patients compared with controls with comparable underlying beating rates [43]. Impaired contractility was associated with reduced mitochondrial content, mitochondrial respiration, and oxidative metabolism in HLHS hiPSC-CMs. These data also suggested that altering the metabolic substrate can improve contractility, thus opening new therapeutic options. Similarly, HLHS-hiPSC-CMs from early heart failure patients showed increased apoptosis, uncompensated oxidative stress, and mitochondrial dysfunction. These abnormalities could be partially rescued by treatment with agents inhibiting mitochondrial permeability transition pore opening or suppressing endoplasmic reticulum stress [43]. Together, these findings provide new evidence that mitochondrial dysfunction could drive not only cellular phenotype of HLHS, but also its disease severity and progression.

Nevertheless, 2D hiPSC-CMs cannot fully reproduce the complex architecture and multicellular environment of HLHS. 3D models replicating the complex heart architecture and dynamic environment represent an important next step for integrating the genetic, metabolic, and developmental properties of human-derived cells. Besides confirming and integrating current knowledge on underlying genetic and pathogenetic mechanisms, these systems will provide new models for new drug testing [29, 44]. For example, cardioids have already been exploited to study the interplay between cardiomyocyte differentiation and cardiac chamber morphogenesis, by KO of *HAND1* and *NKX2-5* genes [36]. Such models provide novel ways to investigate HLHS metabolic pathogenesis and evaluate targeted therapeutic interventions.

### Sarcomere abnormalities: pediatric hypertrophic cardiomyopathy

Hypertrophic cardiomyopathy (HCM) is the most common inherited heart disease, characterized by progressive myocardial hypertrophy, cardiomyocyte disarray, and predisposition to malignant ventricular arrhythmias and heart failure. HCM affects at least 1 in 500 individuals and represents the leading cause of heart failure and sudden cardiac death in children and adolescents [45]. HCM is transmitted in an autosomal dominant fashion, and frequently involves mutations in sarcomeric genes, particularly those encoding beta-myosin heavy chain (MYH7) and myosin-binding protein (MYBPC3), both of which are located in the thick filament of the sarcomere. Many pediatric patients display multiple mutations.

Several models of hiPSC-CMs have been derived to study pathogenetic mechanisms and drug responses, exploiting both patient-derived or CRISPR-edited hiPSCs [46, 47]. However, these studies focused mainly on adult-onset HCM instead of pediatric HCM which is often more severe and has rapidly progressing phenotypes. Recently hiPSCs from pediatric HCM patients were generated and succeeded in recapitulating key disease-associated phenotypes, including cell hypertrophy and sarcomere disorganization, whilst providing a platform to evaluate the efficacy of different drugs, in particular myosin ATPase inhibitors [48]. In addition, hiPSC-derived cardiac tissues (Biowires) enabled the measurement of tissue contractile force and demonstrated pharmacological rescue of abnormal contractility [48]. Together, these models provide an opportunity to link patient-specific variants to functional cardiac phenotypes and evaluate their clinical applications.

Similar to other forms of CHDs, the pathogenesis of sarcomeric HCM also seems to rely on altered myocardial energetics and mitochondrial metabolism. HCM-associated mutations, including those in *MYH7* or *MYBPC3* genes, can increase the energetic cost of contractions and cause inefficient ATP utilization [49, 50]. How mutation-induced energy depletion changes cellular metabolism and increases mitochondrial workload potentially creates a vicious cycle that contributes to HCM disease progression [51]. This mechanism could be better understood by using hiPSC-CM models, as such models may also help elucidate the interplay between metabolic and structural changes during HCM development. Importantly, patient-derived hiPSC-CM models could provide a platform for testing whether early metabolic treatments could restore mitochondrial function and ameliorate HCM disease phenotype.

### Calcium-handling and electrophysiological abnormalities: arrhythmogenic disorders

Ca^2+^ is an important second messenger in cardiac excitation–contraction coupling, where dysfunction in Ca^2+^ handling presents a major cause of arrhythmias and contractile problems even in the absence of any structural cardiac abnormalities [52]. Importantly, Ca^2+^ homeostasis is also tightly linked to mitochondrial metabolism in the heart as mitochondrial Ca^2+^ uptake stimulates energy metabolism to match ATP production with the energetic demands of cardiac contraction, while excessive mitochondrial Ca^2+^ accumulation can activate cell death pathways [53]. Patient-derived hiPSC-CMs provide a particularly valuable platform to simultaneously study Ca^2+^ dynamics, contractility, mitochondrial function, and electrophysiology, all of which are related to arrhythmogenic disorders. Two relevant examples of altered Ca^2+^ handling are calmodulin-associated long QT syndrome (LQTS) and two *RYR2*-associated disorders.

#### LQTS—*CALM* genes

LQTS is an inherited cardiomyopathy that predisposes individuals to life-threatening ventricular arrhythmias. LQTS is mainly associated with defects in genes encoding cardiac potassium and sodium channels (*KCNQ1, KCNH2, SCN5A*). However, a subtype of LQTS is associated with pathogenic variants in three calmodulin-encoding genes (*CALM1-3*) that have also been implicated in sudden infant death syndrome [54].

Calmodulin, a Ca^2+^-binding protein, has a pivotal role in regulating several cardiac ion channels. For instance, calmodulin contributes to the assembly and localization of the Kv7.1 potassium channel, whose loss of function constitutes the most frequent cause of LQTS [55]. Calmodulin also mediates Ca^2+^-dependent inactivation of L-type Ca^2+^ channels, a mechanism that has been investigated using hiPSC-CMs [56]. HiPSC-CMs carrying missense mutations in one of the three *CALM* genes have been generated and shown to recapitulate cellular LQTS phenotypes (including disrupted Ca^2+^ cycling and prolonged action potential duration [APD]), providing a platform to test new therapeutic options. For example, suppression of pathogenic *CALM* expression, using *CALM* genes silencing by CRISPR interference (CRISPRi) has been proposed as it resulted in functional rescue, including normalization of the APD and Ca^2+^/calmodulin-dependent inactivation [56]. Furthermore, short hairpin RNAs have been used to selectively suppress the expression of mutant *CALM* genes in patient-derived hiPSC-CMs. This proof-of-principle strategy, called “suppression-replacement gene therapy”, turned out to be able to revert the *in vitro* disease phenotype by shortening the prolonged APD [57]. Together, these findings show how mutations in *CALM* genes can disrupt Ca^2+^-dependent inactivation of L-type Ca^2+^ channels, resulting in disrupted Ca^2+^ handling and prolonged APD. This causes arrhythmogenic phenotypes that can be rescued by targeted suppression of pathogenic *CALM* gene variants.

#### Catecholaminergic polymorphic ventricular tachycardia/calcium release deficiency syndrome—*RYR2* gene

The *RYR2* gene encodes RyR2, a Ca^2+^ channel located on the membrane of the sarcoplasmic reticulum. RyR2 has a pivotal role in Ca^2+^ release into the cytosol and cardiac excitation–contraction coupling. Noteworthy, RyR2-mediated Ca^2+^ signaling directly affects mitochondrial energy metabolism by helping to match mitochondrial energy supply with cardiac demand. More precisely, experimental reduction of cardiac RyR2 resulted in reduced mitochondrial Ca^2+^, oxidative metabolism, and ATP production [58]. Accordingly, *RYR2* dysfunction seems to have consequences in mitochondrial energy metabolism, providing an important link between arrhythmogenic disorders and metabolism.

Mutations in the *RYR2* gene have been associated with two early-onset inherited arrhythmogenic disorders characterized by predisposition to life-threatening ventricular arrhythmias in the absence of overt structural anomalies: catecholaminergic polymorphic ventricular tachycardia (CPVT) and calcium release deficiency syndrome (CRDS) [59]. Young patients typically experience syncope or cardiac arrest, often in association with physical exertion or acute emotional stress.

In CPVT, pathogenic *RYR2* variants lead to Ca^2+^ leakage from the sarcoplasmic reticulum, increasing cytoplasmic Ca^2+^ levels, thus triggering arrhythmias through delayed afterdepolarizations. Many different patient-derived hiPSC-based models of CPVT have been able to reproduce key disease phenotypes, showing increased cytosolic Ca^2+^ concentration and altered electrophysiological properties resembling CPVT [60–62]. Several patient-derived CPVT-hiPSC-CMs models have also been exploited to test different antiarrhythmic drugs, such as carvedilol, flecainide, and the tetracaine derivative EL20, some of which successfully abolished Ca^2+^ abnormalities [63, 64]. This opens new avenues for testing the CPVT drugs on hiPSC-CMs.

In contrast to the excessive Ca^2+^ release observed in CPVT, loss-of-function mutations in *RYR2* that are associated with CRDS lead to decreased Ca^2+^ release. This leads to Ca^2+^ accumulation in the sarcoplasmic reticulum with abnormally late systolic Ca^2+^ release events with an increased propensity to early afterdepolarizations that may trigger ventricular arrhythmias [65]. Unlike CPVT, CRDS-associated arrhythmias are generally not induced during conventional exercise stress testing or adrenaline challenge, making clinical diagnosis particularly challenging [66]. Since CRDS is only a recently recognized clinical entity, no cellular models have been developed to date. Patient-derived CRDS-hiPSC-CMs could therefore provide valuable information on the consequences of *RYR2* loss-of-function variants causing CRDS and elucidate its opposing functional effects observed in CPVT.

Altogether, these opposing disorders offer a unique example of how different functional alterations of the same Ca^2+^-handling protein can lead to distinct arrhythmogenic phenotypes, emphasizing the value of hiPSC-CMs for exploring their underlying mechanisms. Moreover, an overlapping link between Ca^2+^ handling and mitochondrial energy metabolism provides additional dimensions that could be investigated using patient-derived hiPSC-CMs.

## Limitations of hiPSC and organoid models on Earth

While human hiPSC models have transformed our ability to study mitochondrial and cardiac disorders, several limitations constrain their full potential when used solely under terrestrial *in vitro* conditions. These challenges span cellular immaturity, genetic and experimental variability, organoid heterogeneity, and incomplete representation of the physiological tissue environment.

### Cellular, metabolic, and electrophysiological immaturity

One of the most persistent limitations of hiPSC-derived models, particularly neurons and cardiomyocytes, is their relative immaturity. For example, hiPSC-CMs typically exhibit immature structural and functional properties, resembling phenotypically and functionally fetal cardiomyocytes, which is reflected in altered gene expression, reduced sarcomere organization, aberrant morphology, and fewer T-tubules [67]. Despite advances in differentiation protocols, hiPSC-derived cells often retain a fetal-like metabolic and gene expression profile, relying heavily on glycolysis rather than OXPHOS. Their mitochondria remain relatively immature, characterized by lower abundance, predominantly perinuclear organization, and rudimentary cristae architecture [15, 68–70]. These characteristics undermine their utility in studying diseases where mitochondrial respiration, OXPHOS pathways, and energy balance are central.

Their electrophysiological properties also remain immature. Compared with mature cardiomyocytes, hiPSC-CMs exhibit lower expression of Ca^2+^-handling proteins, delayed Ca^2+^ release, slower action-potential upstroke velocity, and a less negative resting membrane potential [69]. Their genetic expression also reflects an immature electrophysiological profile, with lower expression of the genes related to electrophysiological function (e.g. *KCNJ2, KCND3, KCNJ8, KCNH7, SCN5A, HCN4, GJA1, JPH2*) [71].

These maturation limitations are particularly relevant to metabolic diseases discussed in this review. In arrhythmogenic disorders such as LQTS, CPVT, and CRDS, related to abnormal Ca^2+^ handling, hiPSC-CMs with immature Ca^2+^ handling and APs could influence the electrophysiological phenotypes attributed to disease-associated variants. Similarly, the predominantly glycolytic metabolism and immature mitochondrial phenotype of hiPSC-derived cells could complicate the interpretation of metabolic abnormalities seen in mitochondrial disorders, HLHS, or TOF. In structural CHDs (TOF and HLHS), cellular immaturity represents an additional challenge as hiPSC-CMs cannot fully reproduce multicellular processes connected to cardiac morphogenesis.

Nevertheless, cellular immaturity is not necessarily disadvantageous for all applications. Many CHDs and severe mitochondrial disorders originate during embryonic development, so the fetal-like state of hiPSCs still provides a valuable model for investigating early developmental disease mechanisms. However, it is important to be aware of the required level of maturation in relation to the developmental stage and specific disease phenotype of interest.

### Genetic, epigenetic, and experimental variability

In addition to cellular immaturity, hiPSC-based models can substantially vary in their genetic, epigenetic, and experimental background. Donor-derived hiPSC lines differ in their genetic backgrounds, which can influence differentiation and cellular phenotypes, and therefore confound comparisons between patient and control cell lines [72]. One approach to minimize this variability is the use of isogenic control lines generated through genome editing. For example, disease-associated variants in patient-derived hiPSCs can be corrected with CRISPR/Cas9 editing while preserving the same genetic background, allowing for much stronger evidence for the variant of interest to contribute to the pathogenic phenotype causally [72].

Another concern for cell line variability is that reprogrammed cells may retain epigenetic signatures from their somatic cells of origin, potentially influencing their differentiation behavior [73]. Furthermore, experimental factors, including culture conditions, non-unified protocols, and passage numbers can contribute to inter-line variability and creation of the batch effect [72]. One particularly relevant example for modeling metabolic disorders is oxygen tension. Conventional cell cultures are commonly maintained near the atmospheric oxygen levels (∼20% O_2_), instead of physiological oxygen levels (∼5% O_2_). Physiological oxygen concentrations have been shown to reduce mitochondrial oxidation and increase glycolytic flux, indicating that the amount of oxygen in the culture could change the balance between glycolysis and OXPHOS on its own [9, 74]. Therefore, metabolic phenotypes observed *in vitro* may be influenced by the experimental environment, rather than disease-associated genotype, emphasizing the importance of using relevant experimental conditions.

### Organoid heterogeneity

The use of 3D organoids offers many advantages over 2D cultures by enabling cell–cell interactions and reproducing certain aspects of tissue organization and development. However, their self-organizing nature introduces additional sources of variability. Organoid differentiation is highly heterogenous; therefore, reproducibility is one of the major challenges. Different experimental batches can differ in their overall shape, maturation, cellular composition, and spatial organization [75, 76]. In, e.g. cardiac organoids, it has been shown that organoid size affected cellular properties in the cardiac organoids, affecting the transcriptomic profile [77]. Such heterogeneity can complicate comparisons between the experimental groups, overshadowing real disease-associated differences. Greater standardization between different approaches in generating organoids is therefore required to improve reproducibility across organoid studies.

### Lack of other physiological contexts in organoid studies

Finally, even advanced organoid models reproduce only part of the complex physiological context of the human body. Elements such as blood flow, hormonal regulation, immune interactions, mechanical loading, and multi-organ interactions are crucial in diseases where mitochondrial dysfunction manifests in a systemic or multi-organ manner [78].

For instance, most cardiac and cerebral organoids lack proper vascularization, which prevents their proper function and growth. As organoids increase in size, diffusion alone becomes insufficient to provide oxygen and nutrients and remove metabolic waste from their interior, potentially resulting in necrotic regions [75]. Several approaches have therefore been attempted to induce vascular-like structures in metabolically demanding organoid models, including those representing the brain, heart, kidney, and liver [75].

Organoids may also insufficiently reproduce the multicellular composition of native tissues, such as lack of stromal components in cardiac organoids or microglia in neural organoids. This is important because supporting cells can be involved in metabolic and functional pathological phenotypes. For example, introducing cardiac endothelial cells and cardiac fibroblasts into 3D cardiac microtissues can improve sarcomere structure, contractility, electrophysiology, and mitochondrial respiration [79]. Likewise, in brain organoids, co-culture of neurons and astrocytes also improves the metabolic features and interactions found in the human brain which is not seen in culturing the neurons alone [80]. Thus, incomplete cellular composition in organoids influences not only tissue architecture, but also its metabolic and physiological phenotypes.

Collectively, cellular maturation state, genetic background, culture conditions, organoid heterogeneity, and cellular composition represent substantial limitations when interpreting findings from hiPSC-derived cells and organoids, as each of these factors can independently influence metabolic, mitochondrial, and physiological phenotype, potentially confounding changes associated with the disease pathology of interest. Advances in isogenic gene editing, standardized protocols, multicellular co-cultures, and vascularized organoids are progressively addressing these limitations, thereby improving the physiological relevance, reproducibility, and translational value of hiPSC-based disease models.

Importantly, the International Space Station (ISS) offers a unique platform that may help address some of the aforementioned limitations. By exposing cells to microgravity, space radiation, and altered fluid dynamics, the ISS enables the study of environmental stressors that are difficult to reproduce under terrestrial culture conditions. In particular, microgravity alters mechanotransduction, cytoskeletal tension, and intracellular force balance, whereas space radiation contributes to DNA damage responses and oxidative stress through high linear energy transfer particles [81]. Distinguishing the effects of these individual spaceflight factors is therefore important for interpreting mitochondrial and cardiac phenotypes observed in orbit.

## The ISS: a unique laboratory to study mitochondrial and metabolic dysfunction

The ISS laboratory has investigated a wide range of biological problems, providing insights into various processes relevant to mitochondrial function, cytoskeleton impairment, oxidative stress, and protein catabolism. It has been an invaluable environment to study cellular behavior and uncover notable changes in biochemical, metabolic, genetic, and cellular pathways, many of which overlap with processes observed in diseases on Earth [2]. Space environment conditions appear to shift the balance between physiology and pathophysiology, accelerating musculoskeletal and neurological alterations that resemble aspects of the progression of certain diseases on Earth. Gravitational forces normally regulate cell and tissue homeostasis, since mechanotransduction enables cells to convert mechanical inputs into biochemical signals. However, this process is disrupted in spaceflight, leading to changes in cell shape and cytoskeletal organization [82]. Thus, research in the space environment can rapidly induce phenotypes, such as muscle atrophy and bone loss, facilitating the understanding of their cellular processes that develop more gradually on Earth, and therefore help with identification of potential drug targets [2].

A large body of research has shown that the space environment affects the respiratory chain and metabolic pathways, triggering the generation of ROS through premature electron leakage from the electron transport chain. The imbalance between ROS production and antioxidant defense in mitochondria can ultimately lead to mitochondrial dysfunction [4]. This is especially relevant to mitochondrial disorders and CHDs, both of which involve alterations in mitochondrial metabolism and oxidative stress [6, 7, 83]. Moreover, mitochondrial dysfunction is also interconnected with other cellular responses implicated in spaceflight, including altered protein homeostasis [83]. Protein catabolism in children, including increased protein degradation and reduced protein synthesis, is a major determinant of several clinical syndromes, such as severe under-nutrition/malnutrition, sarcopenia, and systemic response to burns, all of which can lead to muscle mass loss and impaired muscle mitochondrial processes. Likewise, altered protein homeostasis during spaceflight triggers weight and fluid changes, musculoskeletal loss, and muscle atrophy [83].

Related to metabolic changes, microgravity-induced cytoskeletal changes are also noteworthy due to the close interaction between cellular metabolism, mechanotransduction, and tissue development. Several missions have widely investigated the changes in the cytoskeleton under microgravity, including programs developed by the National Aeronautics and Space Administration (NASA) [82]. To date, most research focused on these mechanisms has been conducted in missions using rodents to simulate an accelerated disease model and to test potential countermeasures [1, 4]. The use of hiPSC-derived models complements these studies by enabling microgravity-induced cytoskeletal and metabolic changes to be observed directly in human cells with disease-relevant cell types and genetic background.

Besides disease modelling, the 3D cell culture systems developed in space can sometimes support greater cell viability and functionality (for specific cell states), which may be crucial for regenerative medicine and tissue engineering. Their scalability also makes them ideal for biomanufacturing therapeutic cells and organoids in microgravity conditions. These advancements offer significant therapeutic potential and open new avenues for personalized medicine, wherein patient-specific spheroids can be used to tailor treatments [84]. This is particularly beneficial in patient populations with rare and heterogenous conditions, since they often have limited patient populations which makes large clinical studies difficult. Similarly, diverse genetic backgrounds can substantially influence disease phenotypes and treatment responses, strengthening the need for patient-derived cell models.

## In-space stem cell models: from disease modeling to potential therapeutics

Currently, disease modeling in a space environment represents one of the most important research clusters of bioproduction or biomanufacturing in space [85], since it could speed up cell manufacturing in a way that is not possible on Earth, and commercial systems have already begun to emerge (e.g. Lambdavision for space-grown retinas). Growing cells in space enables the formation of 3D cell culture systems that offer significant advantages over traditional 2D systems by more closely replicating the natural environment of human tissues and enhancing cell–cell and cell–matrix interactions. These cultures may consequently provide more predictive models for evaluating drug efficacy and toxicity [84]. Below, we discuss recent advances in space-based hiPSC research (Table 1) and their potential to advance our understanding of metabolic disorders, particularly mitochondrial diseases and CHDs, which share several molecular signatures with spaceflight-induced cellular stress (Fig. 2).

**Figure 2:**
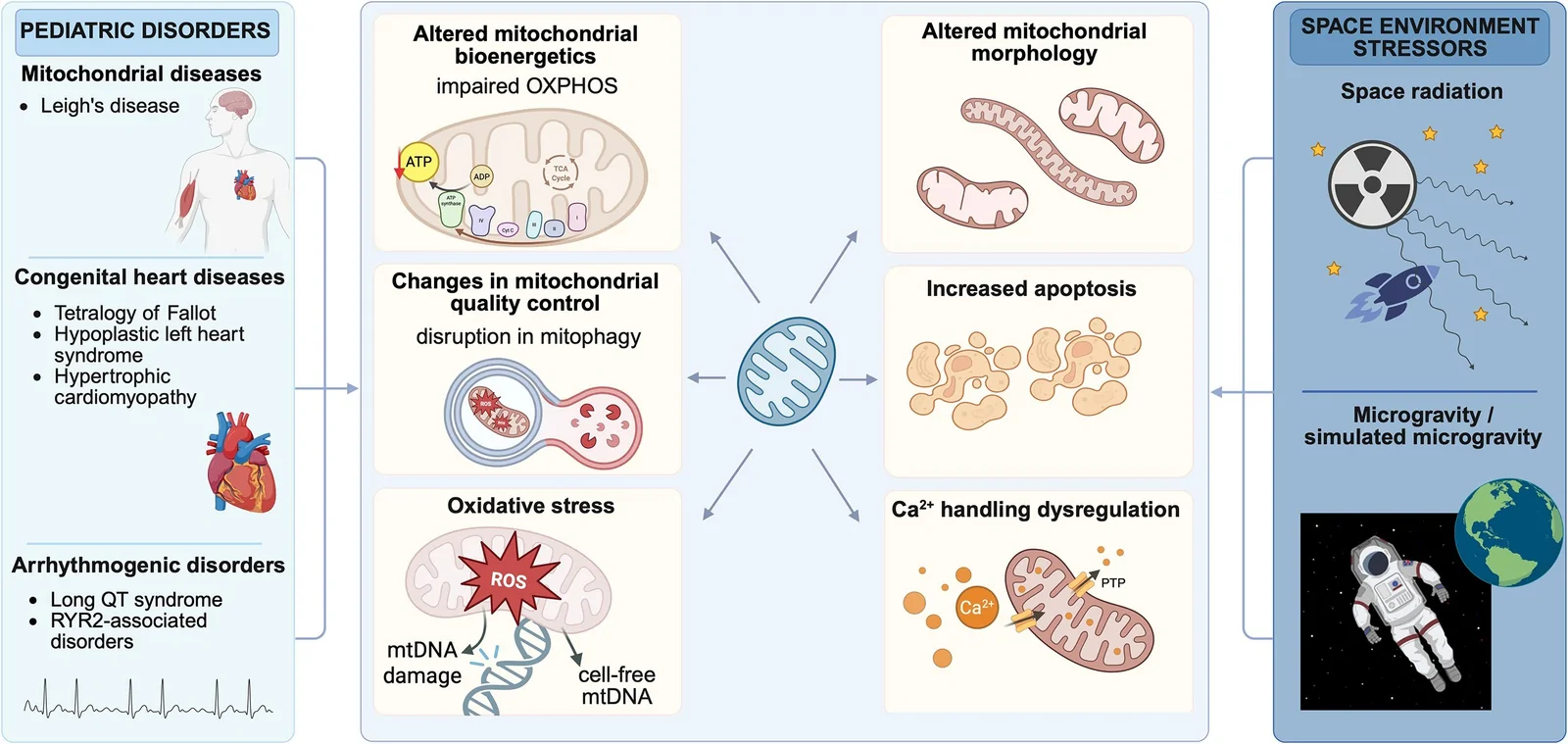
Shared molecular and metabolic signatures between spaceflight-induced stress and mitochondrial diseases and congenital heart disorders based on findings from hiPSC studies. Pediatric disorders, including mitochondrial diseases, CHDs, and arrhythmogenic disorders, exhibit cellular, metabolic, and molecular abnormalities that overlap with those induced by spaceflight stressors, particularly microgravity and space radiation. This overlap largely converges on mitochondrial processes, including altered mitochondrial bioenergetics characterized by impaired OXPHOS and reduced ATP production. Disrupted mitochondrial quality control with dysfunctional mitophagy may further promote the accumulation of unhealthy mitochondria and contribute to altered mitochondrial morphology. Oxidative stress and increased ROS production can promote mtDNA damage and the release of cell-free mtDNA. Collectively, these mitochondrial disturbances may also promote apoptosis. Lastly, Ca^2+^ handling dysregulation, including altered Ca^2+^ influx and prolonged Ca^2+^ transients, can further contribute to pathological phenotypes such as arrhythmia. PTP, permeability transition pore. Created with Biorender.com.

**Table 1:** Summary of available studies using hiPSC-derived models under spaceflight or simulated microgravity conditions, highlighting metabolic, mitochondrial, developmental, and functional responses relevant to disease modeling and translational applications.

Mission/platform	Stem cell type	Conditions	Duration	Key findings	Limitations	Study
Space X CRS-9 mission (ISS)	hiPSC-CM generated from PBMC-derived hiPSC lines	Real spaceflight conditions,	5.5 weeks aboard the ISS	2 635 differentially expressed genes among flight, post-flight Enrichment of pathways related to mitochondrial function/metabolism Enriched transcription factor motifs regulating cardiac metabolism Altered Ca^2+^ handling (decreased Ca^2+^ recycling rate, increased beating irregularity after return) Changes in genes associated with myofilament function No changes in contractility, cell morphology, or sarcomere structure	Different RNA preservation time points between flight, post-flight and ground samples Absence of an on orbit 1*g* centrifuge control Flight samples experienced higher radiation exposure than ground controls Launch and re-entry forces could affect gene expression and cellular phenotypes	[86]
Shenzhou-13 mission, Tianhe core module, CSS	hiPSC-CM generated from renal epithelial cells of a healthy adult and H9 hESC-derived GCaMP-CMs (to study Ca^2+^ signaling)	Real spaceflight conditions (microgravity [μg] compared with an on-orbit simulated 1 g centrifuge control)	144 h (6 days) on orbit	Metabolic reprogramming (especially thiamine metabolism) Reduced TCA flux/downregulation of genes involved in pyruvate oxidation Reduced ATP production OXPHOS defects in simulated microgravity Reduced beating rate/prolonged Ca^2+^ recycling Cytoskeletal/sarcomeric remodelling Thiamine supplementation or SLC19A2 overexpression partially rescued metabolic and functional alterations	Short-term flight data only hiPSC-CMs present only single-cell type and were relatively immature Lack of multicellular/tissue-level interactions	[87]
Ground-based clinostat	hiPSC-CMs	SMG (though slide-flask 2D-clinostat device); compared with 1 *g* control conditions	48 h	Increased ROS production Impaired mitochondrial membrane potential Changed mitochondrial morphology (hyper-fusion of mitochondria) Induction of cellular senescence Contractile dysfunction/reduced Ca^2+^ flux Transcriptomic and proteomic changes related to metabolism (upregulated expression of the antioxidant genes, genes related to cardiomyocyte atrophy) Cytoskeletal remodelling	Immaturity of hiPSC-CMs Short exposure to microgravity Validation under real microgravity conditions needed (e.g. ISS)	[88]
SpaceX Crew-8 mission, ISS	3D hiPSC-CMs (spheroids)	Real spaceflight conditions (control cells cultured in the same cell culture conditions)	8 days	Upregulation of 90 metabolism-associated proteins and 62 proteins associated with mitochondrial cellular component Enriched metabolic pathways related to glutathione and pyruvate metabolism, biosynthesis of amino acids Upregulated proteins related to cellular stress response and cell survival Upregulated genes associated with cardimyocyte development, cellular components of cardiac structure and mitochondrion Normal cell contraction and Ca^2+^ transient parameters	Short-term spaceflight exposure Mitochondrial function was not directly assessed Upstream and downstream signaling mechanisms underlying the metabolic remodelling remain to be determined Molecular analysis was performed after the return to Earth, presenting limited data on transient in-flight responses	[89]
Ground-based random positioning machine (RPM)	3D hiPSC-CMs (cardiac spheres)	SMG (control cells cultured under standard gravity)	7 days (d21–28) or 14 days (d14–28)	Increased mitochondrial content, mitochondrial membrane potential, mitochondrial respiration, and ATP production Improved Ca^2+^ handling (higher Ca^2+^ transient amplitude, faster kinetics of Ca^2+^ transients Structural maturation (increased sarcomere length, z-disc length, nuclear diameter, nuclear eccentricity)	RPM-generated SMG cannot fully recapitulate real spaceflight conditions Effects may depend on SMG exposure time and differentiation stage (different metabolic outcomes on day 7 versus day 14) Contribution of maturation medium effects requires further investigation	[90]
Ground-based clinostat	hiPSC-derived midbrain dopaminergic (DA) organoids (SH-SY5Y DA neurons used as a complementary 2D model)	SMG (1 *g* controls)	72 h (iPSC-derived DA organoids matured for approximately 1 month before exposure)	Reduced mitochondrial membrane potential Suppressed mitochondrial respiration Global translational repression Selective release of cf-mtDNA without apoptotic activation Suppression of mitochondrial metabolism and altered RNA splicing (proteomics) Elevated PINK1/PRKN/LRRK2 signal (increased activation of PD-associated mitochondrial pathways) Selective loss of OXPHOS complexes I, II, and IV Reduced neurite outgrowth Loss of dopaminergic identity	Lack of real spaceflight conditions Mechanistic link between altered mechanical loading and mitochondrial dysfunction remains unknown SMG influence on cellular aging remains unresolved	[91]
SpaceX-19 mission (ISS)	hiPSC-derived cortical and dopaminergic neural organoids from individuals with PPMS or PD and non-symptomatic controls; combined with isogenic microglia	Real spaceflight conditions (ground controls included)	30 days	Lower expression of cell proliferation-associated genes Higher expression of neuronal maturation-associated genes Dopaminergic organoids showed 1183 DEGs and cortical organoids showed 926 DEGs Organoids remained viable after return to Earth and demonstrated neurite outgrowth Little evidence of stress, inflammation, or apoptosis in Earth and LEO samples	Individuals with PPMS and PD had sporadic disease without known genetic basis Mechanisms underlying the apparent accelerated neuronal maturation remain unknown Mitochondrial function/metabolism was not directly assessed	[8]
SpaceX-20 mission (ISS)	3D cardiac progenitor spheres derived from IMR90 hiPSCs	ISS microgravity (μG) vs ISS 1*g* centrifugation control; cultures were thawed and cultured aboard the ISS	22 days	RNA-seq analysis: 470 DEGs between μG and ISS 1*g* conditions Upregulated genes associated with cell cycle, proliferation, cardiac differentiation, development, contraction, and conduction Upregulated pathways associated with cardiac function and adrenergic signaling Upregulation of Ca^2+^ signaling genes (e.g. *ATP2B4, RYR2*) Downregulated genes associated with extracellular matrix regulation	Small sample size (*n* = 3 cultures per condition) Greater number of replicates would be necessary to determine the functional implications of the identified DEGs 3D cultures consisted predominantly of cardiac cells, where their interactions with non-cardiac cells remain unknown Limited in-flight imaging and characterization	[5]
SpaceX-20 mission (ISS)	hiPSC-CMs derived from human iPSC-derived cardiac progenitor cells (CPCs)	ISS microgravity (μG) vs ISS 1*g* centrifugation control and ground controls	3 weeks aboard the ISS	Increased expression of genes associated with proliferation, cardiac development, cell cycle, survival, and cardiac functions Downregulation of genes associated with extracellular matrix, inflammation, cellular stress, and apoptosis Increased expression of genes encoding cardiac structural and Ca^2+^ handling proteins Cardiac spheres cultured in μG were bigger in size and had clearer sarcomere structure Increased peak amplitude and faster kinetics of Ca^2+^ transients	Better flight hardware with better resolution and automatic imaging would be desirable Further functional studies are needed to determine whether transcriptomic changes translate into altered cardiomyocyte function	[92]

Abbreviations: CRS, Commercial Resupply Services; PBMC, peripheral blood mononuclear cell; 1*g*, Earth-normal gravity; RNA, ribonucleic acid; CSS, China Space Station; GCaMP, Genetically encoded Calcium Modulating Protein; hESC, human embryonic stem cell; TCA, tricarboxylic acid; SMG, simulated microgravity; RPM, random positioning machine; DA, dopaminergic; cf-mtDNA, cell-free mitochondrial DNA; PD, Parkinson’s disease; PPMS, primary progressive multiple sclerosis; DEG, differentially expressed gene; RNA-seq, RNA sequencing; CPC, cardiac progenitor cell.

### hiPCS models of mitochondrial diseases in space

Although specific animal models have been developed for mitochondrial disorders, many have limited ability to recapitulate human disease phenotypes [16, 93, 94]. Patient-derived hiPSC models provide a valuable alternative approach, since they allow the study of a specific cell type generated directly from the patients, therefore preserving the relevant genetic background [15, 17–20]. Nevertheless, conventional terrestrial culture conditions may often fail to reveal mitochondrial disease phenotypes. Most notably, due to immature phenotypes of hiPSCs with less defined cristae and energy generation through glycolysis, they show diminished reliance on mitochondrial-based ATP production [15, 68–70]. Therefore, exposing patient-derived hiPSC models to increased metabolic and environmental stress presented by the space environment could provide additional experimental dimensions for investigating the mechanisms present in the mitochondrial diseases.

The space environment provides several stressors that directly affect mitochondrial function, including microgravity and space radiation. Spaceflight has been shown to alter cellular architecture, cytoskeletal organization, and metabolic regulation, where NASA’s GeneLab and omics studies highlighted spaceflight-induced alterations in mitochondrial gene expression, ROS homeostasis, OXPHOS, membrane potential, and ATP production even in otherwise healthy cells and tissues [4]. Space radiation within the space environment may further contribute to mitochondrial stress through ROS production and downstream oxidative damage [95]. These findings imply that mitochondrial dysfunction is a core cellular stress response to the space environment. In this context, the space environment may unmask mitochondrial disease phenotypes in hiPSC-derived cells and accelerate disease processes, enhancing their detectability and mechanistic resolution. Although there are currently no published studies specifically examining hiPSC models of mitochondrial disorders in space, several hiPSC-based studies have assessed mitochondrial and metabolic responses after exposure to real or simulated spaceflight conditions (Fig. 3).

**Figure 3:**
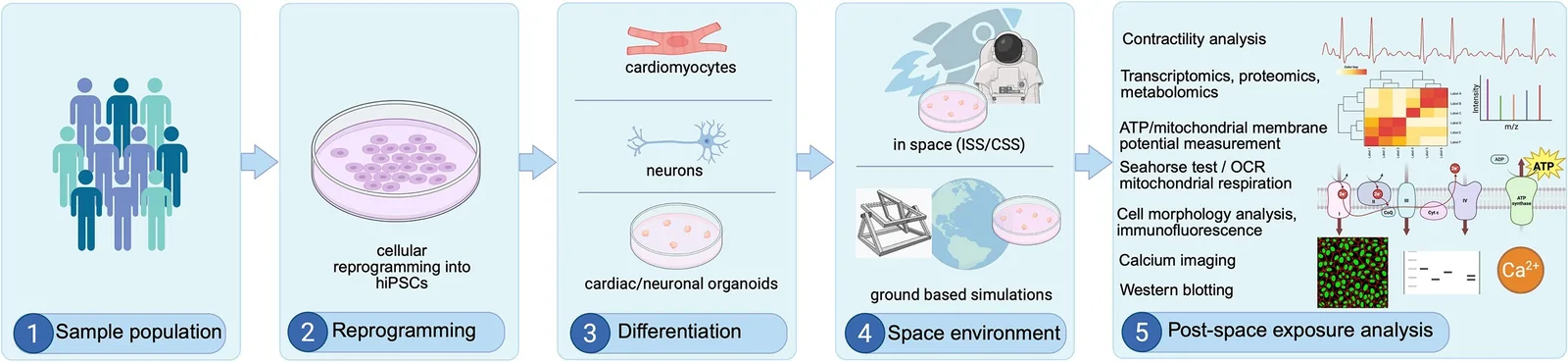
General experimental pipeline of hiPSC-based studies investigating the effects of the space environment. (1) Biological samples are obtained from the study population, (2) donor-derived cells are reprogrammed into hiPSCs, (3) hiPSCs are differentiated into relevant cell types, including cardiomyocytes and neurons, or used to generate cardiac or neuronal 3D organoids, (4) the resulting cell models are exposed either to real spaceflight conditions aboard the ISS or China Space Station (CSS), or to ground-based simulations of spaceflight conditions (e.g. random positioning machine [RPM], clinostat), and (5) cellular responses are characterized using functional, molecular, and metabolic measurements, including contractility measurements, omics approaches, ATP and mitochondrial membrane potential measurements, mitochondrial respiration analysis through oxygen consumption rate (OCR), morphological analysis, immunofluorescence, calcium imaging, and western blotting. Created with Biorender.com.

#### hiPSC-CM-based studies exploring metabolic effects of space

In 2019, Wnorowski *et al*. conducted the first study investigating the effects of spaceflight on hiPSC-CMs and found enrichment of gene-expression pathways related to mitochondrial function in space-flown cells [86]. Similar mitochondrial transcription alterations have been reported across other cell and tissue types exposed to spaceflight [96], suggesting that this response may not be specific to cardiomyocytes. Notably, some mitochondrial gene-expression changes persisted after the cells returned to normal gravity, indicating that spaceflight-induced alterations in mitochondrial homeostasis may persist, at least transiently, following return to Earth [86]. Since then, additional studies using hiPSC-CMs have provided evidence that real or simulated microgravity can substantially alter mitochondrial and metabolic processes, resulting in reduced metabolic flux and ATP production, increased intracellular ROS, and altered mitochondrial morphology [87, 88].

Mitochondrial and metabolic remodeling has also been observed in more complex 3D hiPSC-derived cardiac models. Forghani *et al*. cultured hiPSC-CM-derived cardiac spheroids aboard the ISS and identified enrichment of metabolic and mitochondrial pathways, including glutathione and pyruvate metabolism and amino-acid biosynthesis, together with an increased abundance of proteins involved in cellular stress responses, survival, and metabolism [89]. Although the authors discussed these changes in the context of potentially enhanced cell survival, proliferation, and maturation, mitochondrial function and metabolomic profiles were not directly assessed. Therefore, it remains unclear whether the observed metabolic remodeling represents enhanced maturation or mitochondrial dysfunction. Interestingly, a separate ground-based study by Forghani *et al*. provides more direct evidence of a beneficial metabolic response to simulated microgravity (SMG) [90]. A 7-day exposure of 3D hiPSC cardiac spheres to SMG increased mitochondrial content, mitochondrial membrane potential, and respiration, indicating enhanced metabolic maturation. However, these effects were dependent on the SMG exposure time and developmental stage and were not reproduced in the longer 14-day SMG exposure.

Overall, mitochondrial metabolism changes in space seem highly context dependent rather than uniformly beneficial or detrimental. While SMG may promote mitochondrial maturation, real spaceflight exposes cells simultaneously to other stressors besides microgravity, such as space radiation, launch-associated forces, and other environmental factors, which may elicit distinct metabolic responses. Furthermore, differences in space exposure duration, developmental stage of the cells, and culture configuration may contribute to the variable mitochondrial phenotypes reported across studies. Thus, it remains important that future studies determine which components of the space environment promote adaptive or maturation-associated mitochondrial remodeling responses and which induce pathological mitochondrial stress. This distinction would be especially important when assessing patient-derived hiPSC models with pre-existing mitochondrial defects, such as in mitochondrial diseases.

#### Neuronal hiPSC-based studies exploring metabolic effects of space

Importantly, space-associated alterations in mitochondrial metabolism are not restricted to hiPSC-CMs. Recent evidence from hiPSC-derived midbrain DA organoids demonstrated that SMG induces mitochondrial dysfunction, characterized by loss of mitochondrial membrane potential and suppression of mitochondrial respiration [91]. SMG exposure was also associated with the release of cell-free mitochondrial DNA (cf-mtDNA) in the absence of apoptotic signaling. cf-mtDNA has previously been implicated in aging and neuroinflammatory conditions [97] and has also been found at elevated levels in astronaut blood plasma following spaceflight [98]. Furthermore, transcriptional profiling of DA neurons exposed to SMG revealed activation of mitochondrial quality control pathways, including increased expression of *PINK1, PRKN*, and *LRRK2*. Given the established involvement of PINK/Parkin-mediated mitophagy in Parkinson’s disease (PD), together with the convergence of SMG-induced molecular signatures with those observed in early PD, these findings suggest that altered gravitational loading may expose mitochondrial vulnerabilities relevant to DA neurodegeneration [91, 99].

The feasibility of investigating disease-relevant neuronal hiPSC models under actual spaceflight conditions has also already been demonstrated. DA organoids derived from individuals with PD have already been cultured aboard the ISS, although mitochondrial and metabolic function was not directly assessed in this study [8]. Interestingly, space-flown DA organoids exhibited increased expression of neuronal maturation-associated genes and reduced expression of proliferation-associated genes, suggesting accelerated neuronal maturation in LEO. This observation parallels findings from some hiPSC-CM studies in which microgravity was associated with enhanced cellular or metabolic maturation [89, 90]. One important notion is that the PD organoids were derived from individuals with sporadic disease without known genetic cause, and the study was not designed or sufficiently powered to establish disease-specific differences between PD and control lines [8]. Thus, whether disease-associated genetic backgrounds can modify neuronal responses to the space environment is still to be explored.

Collectively, these studies demonstrate that spaceflight and simulated microgravity can alter mitochondrial, metabolic, and maturation-related properties across cardiac and neural hiPSC-derived models. However, their relevance to mitochondrial disorders remains largely unexplored, as none of these studies specifically examined hiPSCs carrying pathogenic variants responsible for mitochondrial disease. It will therefore be important to investigate how patient-derived hiPSCs carrying pathogenic mitochondrial or nuclear variants affecting mitochondrial function respond to spaceflight-associated metabolic stress. In particular, it remains unknown whether such cells could also exhibit adaptive responses with enhanced maturation similar to those reported in some healthy hiPSC models, or whether pre-existing mitochondrial vulnerability would result in exacerbated bioenergetic dysfunction and cellular damage.

### hiPSC models of congenital heart disorders in space

#### Space-induced cardiac dysfunction and convergence with CHD phenotypes

Cardiac stem cell models (Fig. 3) provide some of the strongest evidence for the effects of the space environment on human stem-cell-derived tissues. In particular, metabolic alterations induced by the space environment in hiPSC-CMs may be highly relevant to CHDs, as patient-derived hiPSC-CMs can exhibit mitochondrial abnormalities that overlap with those observed in non-disease hiPSC-CMs exposed to spaceflight stressors, including defects in mitochondrial respiration, increased ROS, and oxidative stress [29, 86, 87, 88]. These metabolic changes may also affect other processes frequently disturbed in CHDs, such as Ca^2+^ handling [31, 59, 41]. Indeed, several studies have reported that exposure of hiPSC-CMs to the space environment or SMG can induce prolonged Ca^2+^ recycling, increased beating irregularity, reduced beating rate, and contractile dysfunction [86, 87, 88].

Transcriptomic analysis of hiPSC-CMs exposed to the space environment or SMG have also revealed cytoskeletal remodeling [87, 88]. Importantly, defects and changes in cytoskeleton proteins also represent the main determinants of a wide array of rare, heterogeneous, and severe disorders, including CHDs and myopathies [100]. This mechanistic overlap may be particularly relevant to developmental cardiac disorders such as HLHS, in which genetically heterogenous abnormalities converge on cellular processes involved in cardiac myogenesis and cardiomyocyte maturation. Impaired maturation of ventricular cardiomyocytes limits their ability to respond to growth cues, resulting in premature cell cycle exit and increased apoptosis under biomechanical stress in 3D heart structures. Indeed, intrinsic cardiomyocyte defects, including loss of replication and increased apoptosis have been reported in patient-derived stem cell models of HLHS under terrestrial conditions [101]. Similarly, hiPSC-CMs exposed to SMG have shown increased markers of cellular senescence and transcriptional changes associated with cardiomyocyte atrophy [88].

Comparable effects on cytoskeletal organization and cellular stress have also been observed in other cell models exposed to altered gravity, suggesting that these responses are not restricted to cardiomyocytes. An investigation of sarcopenia and disuse atrophy in stem-cell-derived myocytes using a LEO-based platform has shown bone remodeling, orchestrated by actin cytoskeleton and microtubule network dysregulation, which may inhibit the bone morphogenic protein signaling axis, resulting in a loss of bone density [102]. In addition, altered transcription, translation, activation of autophagy, and increased apoptosis have been reported in several cell lines exposed to spaceflight conditions [82, 103].

Together, these observations suggest a potential mechanistic convergence between metabolic dysfunction, cytoskeletal remodeling, impaired cellular growth, and apoptosis. Such interactions might be particularly relevant to CHDs, given that mitochondrial and metabolic impairments, including defective MQC, can disrupt cardiomyocyte homeostasis and contribute to cardiomyopathy and activation of apoptotic pathways [6, 27]. These findings also indicate that spaceflight can induce metabolic, functional, and structural alterations that overlap with cellular processes implicated in CHD pathophysiology. Combining disease-specific hiPSC models with the metabolic and cellular perturbations caused by spaceflight could provide a novel experimental approach to investigate how cells with pathogenic CHD phenotypes respond to altered mitochondrial metabolism, Ca^2+^ signaling, oxidative stress, and mechanical cues under the space environment.

#### Space-induced cardiac maturation and functional adaptation

Although the space environment can induce detrimental effects in hiPSC-CMs, several studies have also reported changes consistent with enhanced cardiomyocyte maturation and more mature molecular and functional phenotypes. Several studies using either SMG or real spaceflight conditions have reported upregulation of genes related to proliferation, cardiac differentiation, contraction, and conduction, together with downregulation of genes associated with inflammation, cellular stress, and apoptosis in both 2D and 3D hiPSC-CM models [5, 89, 92]. Furthermore, exposure to microgravity has been associated with increased expression of genes related to Ca^2+^ handling and signaling, and cardiac structural proteins [5, 90, 92]. Upregulation of pathways associated with adrenergic signaling, which is closely linked to Ca^2+^ signaling and cardiac contraction [104], further suggests enhanced cardiomyocyte differentiation and maturation under microgravity [92]. Moreover, hiPSC-CM-derived spheres exposed to microgravity exhibited features of structural maturation, including increased sarcomere length, while increased spheroid size and cellular proliferation have also been reported [90, 92]. Importantly, several studies reporting maturation-associated effects investigated microgravity in isolation [5, 90, 92]. This distinction may be critical, as the response of hiPSC-CMs to microgravity alone may differ from their response to the combined space environment, which additionally includes factors such as space radiation.

These recent advances suggest that the induction of more mature cardiomyocyte phenotypes could be particularly valuable for investigating the metabolic contribution to CHDs. Terrestrial hiPSC-CMs retain a relatively immature metabolic phenotype characterized by a strong reliance on glycolysis, whereas cardiomyocyte maturation involves a transition toward greater dependence on mitochondrial OXPHOS [15, 68]. Thus, if specific features of the space environment promote metabolic maturation, they could potentially reveal mitochondrial and metabolic disease phenotypes that remain less apparent in immature terrestrial hiPSC-CM models. Yet, the apparently contradictory effects of the space environment on cardiomyocyte maturation and dysfunction require careful consideration. For instance, some studies report impaired Ca^2+^ handling, whereas others show changes consistent with improved Ca^2+^ handling and cardiomyocyte maturation. Defining the experimental conditions and individual spaceflight stressors that promote maturation versus those that induce disease-relevant phenotypes will therefore be essential before extending these approaches to patient-derived CHD models. This may be particularly important because patient-derived hiPSC-CMs could respond differently from non-disease cells. For example, hiPSC-CMs from patients with HLHS already exhibit impaired cardiomyocyte maturation under terrestrial conditions [41]. Whether exposure to the space environment could promote maturation in these cells and thereby improve disease modeling, or instead exacerbate their underlying susceptibility to metabolic and mechanical stress, remains unknown. Direct comparisons of patient-derived CHD hiPSC-CMs with healthy and isogenic controls under well-defined spaceflight conditions could therefore help distinguish adaptive responses from disease-specific vulnerabilities and determine how CHD-associated genetic backgrounds influence cellular responses to the space environment.

## Future directions and current limitations

To understand the effects of space on human metabolic processes implicated in mitochondrial diseases and CHDs, future studies will first need to determine which components of the space environment drive adaptive responses in hiPSCs, such as increased maturity and viability, and which promote pathological phenotypes, such as increased ROS production and mitochondrial dysfunction. As summarized in Table 1, hiPSC studies conducted under spaceflight or simulated spaceflight conditions often show divergent cellular and metabolic responses. However, these studies differ substantially in their experimental conditions, which may contribute to the observed variability. Therefore, future studies should carefully assess the individual and combined effects of different spaceflight stressors on hiPSCs. Two major spaceflight stressors are microgravity and space radiation, which can induce distinct cellular responses and may produce additive or synergistic effects when combined. For example, combined exposure to microgravity and radiation has been associated with increased oxidative stress and enhanced chromosomal aberrations compared with individual exposures [105, 106]. Transcriptomics analyses have further demonstrated opposing effects of microgravity and space radiation on specific molecular pathways, with radiation activating pathways associated with hypoxia, inflammation, DNA repair, and apoptosis, consistent with an ageing-like phenotype, while microgravity preferentially activated pathways associated with metabolism and proliferation [107]. Thus, these two stressors should be investigated both independently and in combination to distinguish their individual effects and interactions.

Studies assessing microgravity alone further indicate that it does not produce uniformly beneficial or detrimental effects. Both promoting and inhibitory effects have been reported, with outcomes varying according to experimental conditions, cell source, and observation time [108, 109]. Exposure duration may be particularly important, as cells have been proposed to gradually adapt to microgravity, with cellular damage decreasing over time in some experimental models [108]. On the other hand, space radiation alone has been associated with increased apoptosis and oxidative stress, as well as delayed DNA repair, DNA damage, and mutations with the potential to contribute to malignant transformation [84, 110]. Understanding radiation-induced effects will become increasingly important for missions beyond LEO, where reduced protection from Earth’s magnetosphere results in greater exposure to space radiation [111].

Future experiments should therefore incorporate comparisons between simulated microgravity, radiation, their combined exposure, and actual spaceflight, together with appropriate ground and in-flight 1*g* controls to determine which environmental conditions promote cellular maturation and which contribute to mitochondrial and cardiac dysfunction. Experiments using on-orbit centrifuges providing a 1*g* control within the same radiation environment or with a radiation-shielded cell growth chamber could assist in differentiating these responses. Understanding these exposure-specific responses will be important before extending the approaches to patient-derived hiPSC models of mitochondrial diseases and CHDs, as their underlying metabolic defects may alter their capacity to adapt to spaceflight stressors and reveal disease-specific vulnerabilities.

Beyond the differential impacts of individual space stressors, future hiPSC studies in space should also focus on underlying mechanistic processes, since the causal contribution of mitochondrial dysfunction to spaceflight-induced cardiac dysfunction remains incompletely understood. Mechanistic studies in space could also provide insights into related metabolic disease mechanisms on Earth, including those underlying CHDs. Most current data presented in Table 1 are based on transcriptional and bioinformatic analyses, with limited mechanistic and functional validation. Future studies should characterize the nature of metabolic impairment and mitochondrial dysfunction and identify the initial factors driving these changes. For example, a recent study by Wakigawa *et al*. demonstrated that mitochondrial impairment observed in space may arise through mechanisms beyond the commonly reported increase in ROS [112]. The authors showed that mitochondria utilize gravity to regulate protein synthesis within the organelle, revealing a previously unrecognized mechanism through which altered gravity can directly influence mitochondrial function. Identifying such mechanisms will be important for interpreting metabolic changes observed in disease models and 3D organoids cultured under microgravity, as well as for distinguishing the initial drivers of mitochondrial dysfunction from their downstream consequences [112].

Future hiPSC studies in space should also address the long-term metabolic changes induced by spaceflight and assess whether metabolic reprogramming persists or returns to its pre-flight state after return to terrestrial conditions. Such studies could help determine how healthy cells adapt to and recover from spaceflight stressors, while comparisons with patient-derived hiPSCs could reveal whether disease-associated defects impair these adaptive responses. For example, patient-derived hiPSCs carrying mutations that impair mitochondrial function may show less-efficient metabolic adaptation or recovery than healthy controls. Identifying where these responses fail could provide new insights into the metabolic vulnerabilities underlying mitochondrial diseases and CHDs.

While promising, ISS-based studies also come with their own set of experimental and logistical challenges. The number of biological samples that can be sent to space is extremely limited, restricting replicability and high-throughput screening. Launch constraints, delayed sample return, and limitations in real-time analysis further reduce experimental flexibility. Constraints on crew time and limited automation, together with challenges in preserving dynamic cellular states during fixation or sample return, further restrict experimental scale and reproducibility. Technical readiness is still evolving, although automation and remote microscopy are improving the feasibility of complex experiments in orbit. Future tissue-chip programs and collaborative initiatives integrating expertise in bioengineering, stem cell technology, organ physiology, pharmacology, toxicology, pathology, and regulatory science could help overcome these limitations and facilitate more standardized and scalable space-based hiPSC studies.

## Integrative framework and clinical applications

For rare and heterogenous disorders such as mitochondrial diseases and CHDs, patient-derived hiPSCs provide particularly valuable disease models because they preserve the diverse genetic backgrounds underlying these conditions. However, several limitations of terrestrial hiPSC models can restrict their ability to reproduce disease-relevant phenotypes. This is particularly important for metabolic processes, as hiPSC-derived cells often rely on immature fetal-like metabolic processes with functionally immature mitochondria [15, 68–70]. Developmental organization is also important for reproducing appropriate tissue organization, especially for understanding structural CHDs, in which abnormal cardiac development contributes directly to disease pathology. Studies conducted under space conditions have reported changes associated with increased maturation in several hiPSC-derived models, including more mature metabolic phenotypes [5, 8, 89, 90,92]. Microgravity has also been proposed to facilitate 3D growth of stem-cell-derived organoids in a state that more closely resembles cellular organization within human tissue [113]. Space-based culture may therefore provide an important complementary environment in which disease-associated metabolic or developmental phenotypes that remain less apparent under conventional terrestrial conditions could be revealed. To date, however, patient-derived hiPSC models of mitochondrial diseases or CHDs have not been investigated in the space environment, representing an important opportunity for future research to uncover novel metabolic mechanisms underlying these disorders.

An additional translational opportunity of space-based research arises from the cellular stress induced by the space environment itself. As discussed throughout this review, spaceflight conditions can alter cellular metabolism, ROS production, Ca^2+^ regulation, electrophysiology, and morphology in hiPSC-derived models [86, 87, 88, 91]. Importantly, several of these responses converge with pathways implicated in mitochondrial diseases and CHDs (Fig. 2). Spaceflight could therefore act as a stress environment that amplifies underlying cellular vulnerabilities, providing an opportunity to identify disease-relevant mechanisms that may be difficult to detect under conventional culture conditions.

The translational value of such discoveries will ultimately depend on their validation under terrestrial conditions. Once a disease-relevant mechanism or vulnerability is identified or amplified in space, its contribution to disease should be confirmed in patient-derived hiPSCs and corresponding isogenic controls on Earth. Shared molecular signatures between spaceflight-induced stress and mitochondrial diseases or CHDs could subsequently be used to identify candidate therapeutic targets and support drug-repurposing approaches, some of which have been observed in recent NASA and commercial missions [114–120]. Candidate interventions could then be tested for their ability to rescue relevant metabolic and functional phenotypes, including mitochondrial respiration, ATP production, ROS accumulation, and Ca^2+^ handling. Promising findings could subsequently be validated in physiologically relevant terrestrial models, including organoids, engineered tissues, or organ-on-chip systems, before progressing toward conventional preclinical and ultimately clinical studies. This provides a translational framework in which space serves as a discovery environment, while disease-specific validation and therapeutic development are performed in clinically relevant terrestrial models.

A distinct translational opportunity also arises from the potential to use microgravity for stem-cell and tissue biomanufacturing. Since microgravity can facilitate 3D growth and enhances particular aspects of cellular organization and maturation, it could provide new avenues for stem-cell-derived tissues for subsequent terrestrial research, drug testing, or regenerative applications [113]. In this context, validation of space-based models following their return to Earth is particularly important. Future studies should validate whether cells and tissues generated under microgravity can retain relevant properties, including their stem-cell characteristics, viability, maturation state, and functional phenotypes when returned to terrestrial conditions.

Together these approaches illustrate that understanding how mitochondria and cardiomyocytes behave in space could have two-fold benefits. First, it may provide insights into novel aspects of mitochondrial and cardiac biology that become apparent only under cellular stress in space conditions. Second, it could support the development of countermeasures for astronaut health, as spaceflight-associated complications, such as cardiovascular and neurological alterations, may be linked to mitochondrial damage [96], providing a potential link between terrestrial disease conditions and astronaut health. From a translational standpoint, stress-amplified phenotypes observed in space could prompt terrestrial drug screening by revealing early mitochondrial vulnerabilities or contractile defects, potentially improving target identification and therapeutic stratification for rare pediatric disorders (Fig. 1).

## Conclusions

As we enter a new era of space-based biomanufacturing and disease modeling, hiPSC-derived platforms represent a promising frontier connecting space-based and terrestrial biomedical research. Organoid-based disease models, particularly those of the brain and heart, provide physiologically relevant systems to assess how mitochondrial pathologies evolve in altered gravity and radiation environments. Current studies demonstrate that spaceflight and simulated microgravity can induce both adaptive and pathological responses, including changes in mitochondrial function, oxidative stress, cytoskeletal organization, Ca^2+^ handling, and cellular maturation. Since these responses converge with key phenotypes observed in mitochondrial disorders and CHDs, spaceflight stressors might reveal or amplify disease-associated phenotypes that are less apparent under conventional terrestrial culture conditions. To date, patient-derived models of rare metabolic disorders have not been investigated under spaceflight conditions, representing an important gap in this field. Future studies integrating patient-derived hiPSCs in space could help identify disease mechanisms and determine their relevance for therapeutic development on Earth. Ultimately, the intersection of stem cell biology and spaceflight holds transformative potential, not only for understanding rare metabolic disorders but also for advancing regenerative medicine, precision medicine, and human adaptation to space.
